# Case Report: Interventricular septal hematoma after ventricular septal defect repair with persistent left ventricular communication: 10-year follow-up

**DOI:** 10.3389/fcvm.2026.1922293

**Published:** 2026-08-27

**Authors:** Anna Rudek-Budzyńska, Aleksandra Morka, Tomasz Mroczek

**Affiliations:** 1 Department of Pediatric Cardiac Surgery, Jagiellonian University, Collegium Medicum, Krakow, Poland; 2 Department of Pediatric Cardiology, Jagiellonian Univeristy, Collegium Medicum, Krakow, Poland

**Keywords:** cardiac magnetic resonance, congenital heart surgery, echocardiography, interventricular septal hematoma, ventricular septal defect, congenital heart disease, ischemic stroke, anticoagulation

## Abstract

Interventricular septal hematoma is a rare complication of congenital heart surgery with unpredictable clinical course and limited data guiding management. We report a female infant who developed a giant interventricular septal hematoma after surgical closure of a perimembranous ventricular septal defect at 4 months of age. Early postoperative imaging demonstrated a large intraseptal cavity without ventricular communication, followed by development of multiple small fistulous connections with the left ventricular lumen one month later. Cardiac magnetic resonance imaging confirmed an extensive blood-filled cavity involving the basal, mid and apical interventricular septum, with preserved global ventricular systolic function. Serial multimodality imaging over 10 years demonstrated stable hematoma morphology and persistent communication with the left ventricle without hemodynamic compromise. Conservative management was maintained; however, ischemic stroke occurred five years after surgery, requiring long-term anticoagulation. This case represents the longest reported follow-up of interventricular septal hematoma with persistent left ventricular communication and provides insight into the natural history of this rare entity, demonstrating that long-term structural stability and preserved ventricular function may be achieved under non-interventional management despite the risk of thromboembolic complications.

## Introduction

Interventricular septal hematoma is an uncommon but potentially life-threatening complication following surgical repair of congenital heart disease (CHD) (1, 2). It has been most frequently described after closure of perimembranous ventricular septal defects (VSDs), repair of tetralogy of Fallot, coronary intervention, myocardial infarction, or myocardial trauma (1–3). In adults, this entity is typically associated with coronary procedures or ischemic injury, whereas in pediatric patients it remains extremely rare (2, 3).

The clinical course is heterogeneous and unpredictable (1–3). While some hematomas undergo spontaneous resolution, others may lead to severe complications including atrioventricular conduction disturbances, ventricular outflow tract obstruction, cardiac tamponade, heart transplantation, or death (1–3).

Septal hematomas without communication to a ventricular cavity usually resolve spontaneously, whereas lesions with communication appear prone to persistence (1, 2).

We present a unique case of a giant interventricular septal hematoma with persistent communication to the LV cavity following surgical closure of a perimembranous VSD, with continuous 10-year follow-up.

### Case presentation

A female infant was diagnosed with a hemodynamically significant perimembranous ventricular septal defect during the neonatal period after evaluation of a cardiac murmur. She underwent elective surgical closure at 5 months of age because of persistent symptoms of heart failure despite standard medical therapy. She was born at term following an uncomplicated pregnancy and delivery, with an uneventful neonatal adaptation, and demonstrated normal growth and psychomotor development before surgery. There was no family history of congenital heart disease, inherited cardiomyopathy, thromboembolic disease, or known coagulation disorders, and no syndromic features or genetic abnormalities were identified. Before surgical repair, the patient had not undergone any previous cardiac interventions. Physical examination before surgery revealed a systolic murmur along the left sternal border with clinical signs consistent with heart failure secondary to a large ventricular septal defect. No cyanosis or extracardiac abnormalities were observed.

The operation was performed under moderate hypothermia (26 °C) via a right atrial approach through the tricuspid valve. Myocardial protection was achieved using standard crystalloid cardioplegia delivered into the ascending aorta with a roller pump under electronically controlled pressure limited to 30 mmHg.

The defect was closed using a Dacron patch with a continuous 5-0 Prolene suture. The aortic cross-clamp and myocardial ischemic time was 35 min. No technical difficulties were encountered intraoperatively.

**Figure 1:**
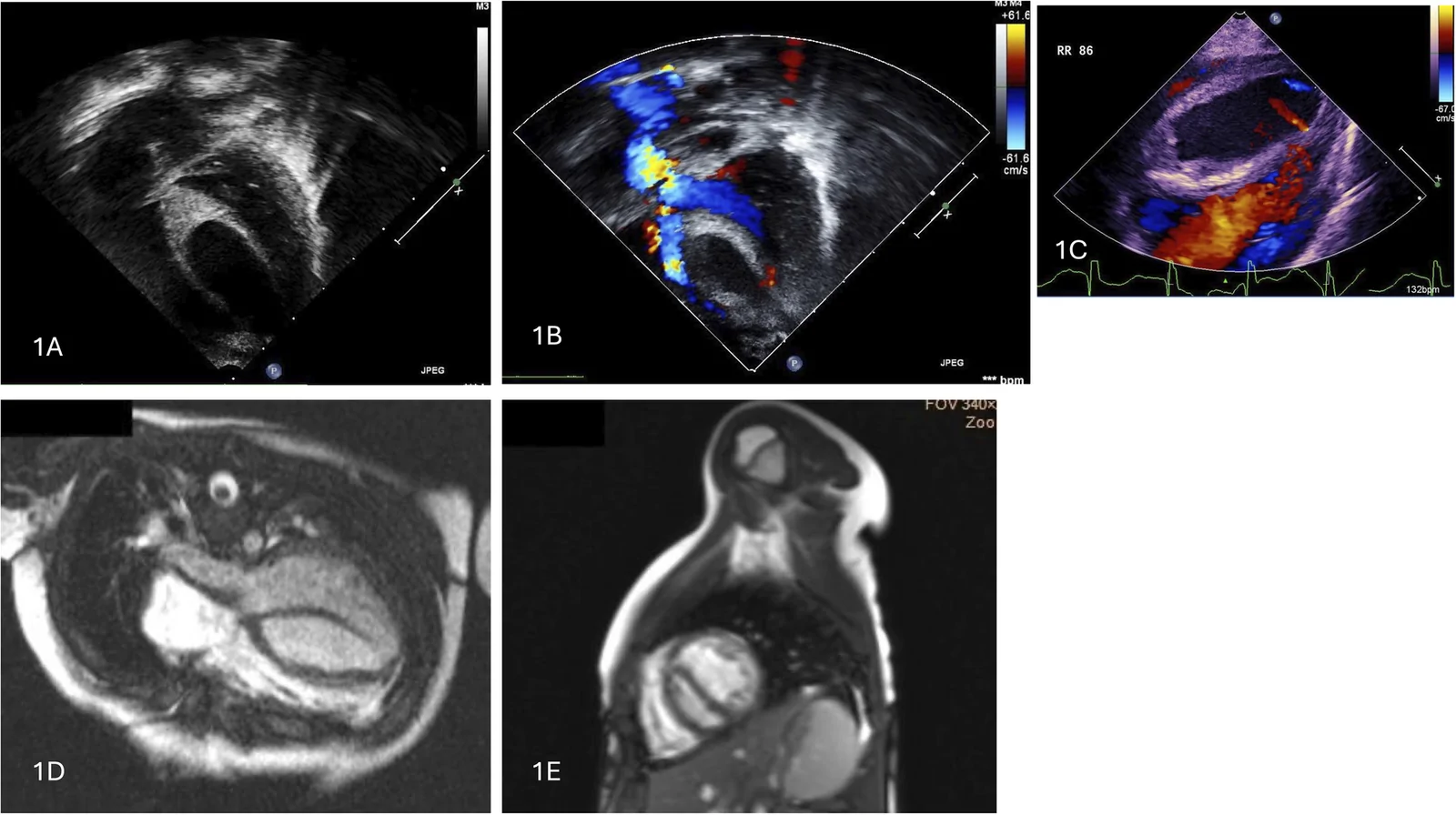
**(A,B)** Transthoracic echocardiography demonstrating a large intraseptal hematoma located within the interventricular septum, protruding toward both ventricular cavities. **(C)** Transthoracic echocardiography showing multiple small fistulous communications between the interventricular septal hematoma cavity and the left ventricular lumen. **(D,E)** Cardiac magnetic resonance imaging demonstrating dissected interventricular septal myocardium with communication to the left ventricular cavity at the apical septal segment.

Immediate postoperative transthoracic echocardiography revealed a fluid-filled intraseptal space (Figure 1A) measuring 28 × 15 mm consistent with an interventricular septal hematoma. The lesion protruded into both ventricular cavities (Figure 1B) but did not compromise inflow or outflow tracts (Supplementary Videos 1–3) No communication between the hematoma and the ventricular cavities was initially detected (Supplementary Videos 3, 4)*.*

Interventricular septal contractility was impaired, whereas systolic function of both ventricular free walls remained preserved. A small residual VSD measuring approximately 1 mm was observed (Supplementary Video 4)*.*

### Postoperative course

The patient required transient vasoactive support with milrinone, dopamine, sodium nitroprusside, and isoproterenol, which were gradually withdrawn without clinical deterioration. Early postoperative rhythm disturbances included first- and second-degree AV block and frequent supraventricular arrhythmias originating near the AV junction. Intensive antiarrhythmic therapy resulted in restoration of stable sinus rhythm.

Serial echocardiography demonstrated no early increase in hematoma size. One month postoperatively, the hematoma enlarged to 30 × 18 mm and extended along the entire interventricular septum. Multiple small fistulous communications between the hematoma cavity and the LV lumen, predominantly in the apical region, became evident (Figure 1C, Supplementary Video 5)*.* Although these communications resulted in partial decompression of the hematoma, neither regression of the lesion nor recovery of septal contractility was observed. No residual ventricular septal defect was identified.

Despite progressive enlargement of the hematoma during the first postoperative month, conservative treatment was continued because the patient remained hemodynamically stable, ventricular systolic function was preserved, and there was no evidence of ventricular outflow tract obstruction or progressive clinical deterioration. Following multidisciplinary consultation, the benefits of conservative management were considered to outweigh the risks of repeat surgical intervention.

Consequently, invasive treatment was deferred, and the patient was discharged home on postoperative day 55 in stable clinical condition under conservative management.

### Advanced imaging and follow-up

Cardiac magnetic resonance imaging performed at 7 months of age demonstrated a large blood-filled cavity measuring 45 × 18 × 42 mm involving basal, mid, and apical segments of the interventricular septum. The hematoma was surrounded by dissected septal myocardium, thinner on the LV side, with persistent communication to the LV cavity at the apical level (Figures 1D,E, Supplementary Video 6)*.*

Septal contraction was asynchronous, and ventricular volumes were mildly reduced, without inflow or outflow tract obstruction.

**Figure 2:**
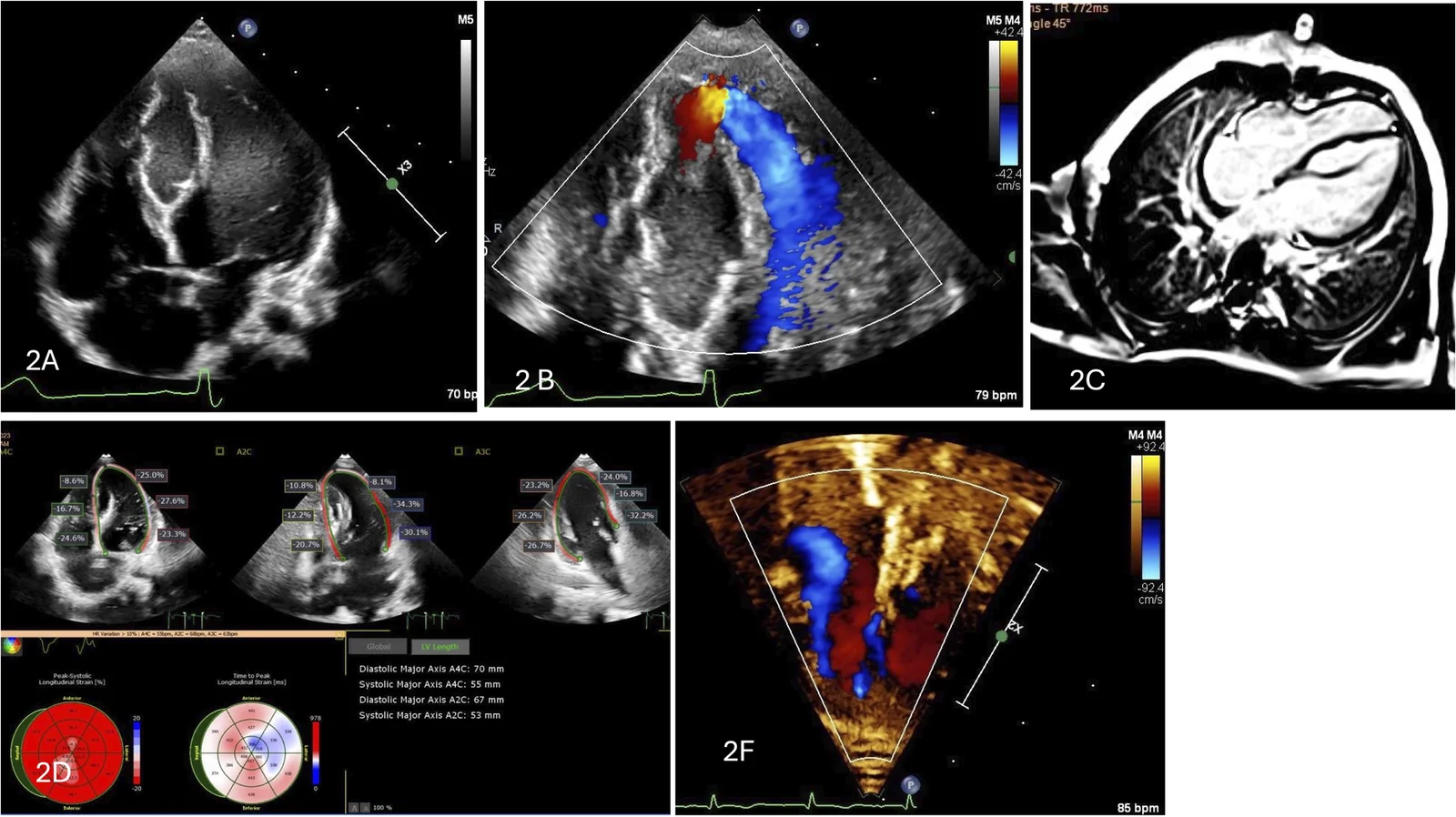
Long-term imaging assessment of a giant interventricular septal hematoma with persistent communication to the left ventricular cavity. **(A)** Transthoracic echocardiography demonstrating a large chronic dissecting cavity within the interventricular septum. **(B)** Color Doppler echocardiography showing persistent communication between the hematoma cavity and the left ventricular lumen. **(C)** Cardiac magnetic resonance imaging (four-chamber view) showing a large blood-filled cavity within the dissected interventricular septum. **(D)** Segmental strain analysis showing localized impairment of septal myocardial deformation with preserved global ventricular systolic performance. **(F)** Transthoracic echocardiography showing hyperechogenic structure suggestive of thrombus formation within the apical portion of the dissected interventricular septal hematoma.

Repeated echocardiographic examinations (initially every 3 months and currently every 6 months) (Supplementary Videos 7–9) and cardiac magnetic resonance imaging (performed at 5 and 9 years) (Figures 2A–D, Supplementary Videos 10, 11) demonstrated excellent concordance and revealed stable hematoma morphology without regression, progression, or new pathological findings during a continuous 10-year follow-up period.

Global systolic function of both ventricles remained preserved apart from localized septal dysfunction and remodeling.

Communication between the hematoma cavity and the LV remained patent throughout the entire observation period with no tendency toward spontaneous closure.

### Late complications

Five years after surgery, despite antiplatelet therapy (Aspirin 2 mg/kg), the patient experienced an acute neurological episode characterized by sudden onset of left-sided hemiparesis, more pronounced in the upper limb, accompanied by speech disturbance and transient impairment of consciousness without loss of consciousness. The symptoms occurred abruptly following a period of normal activity and previously stable clinical condition.

Neuroimaging (CT scan, MRI, angio MRI) revealed an ischemic stroke involving the right cerebral hemisphere, affecting the temporal region and subcortical structures, consistent with the observed left-sided neurological deficits. The imaging findings demonstrated evolution of the lesions from the acute to the subacute phase, followed by the development of chronic post-ischemic changes, including cavitation and gliosis. No abnormalities of the cerebral vasculature or aneurysms were identified, and no peripheral thromboembolic sources or coagulation disorders were detected.

Transthoracic echocardiography demonstrated preserved cardiac function and a stable appearance of the interventricular septal hematoma. However, within the apical portion of the lesion, a hyperechogenic structure was identified, partially limiting inflow to this region and potentially corresponding to evolving thrombus formation (Figure 2E)*.*

### Diagnostic assessment and management

The diagnosis of interventricular septal hematoma was established based on transthoracic echocardiography and subsequently confirmed by cardiac magnetic resonance imaging. Conservative management strategy was adopted in the absence of hemodynamic compromise. Invasive intervention was deferred due to risk of atrioventricular conduction disturbances and potential continuation of intramyocardial dissection. Following ischemic stroke, oral anticoagulation therapy was initiated. Neurological deficits largely resolved with rehabilitation. The patient is currently treated with angiotensin-converting enzyme inhibitor therapy and remains on oral anticoagulation. She demonstrates normal overall development and participates in social activities with some limitations. The girl attends school and takes part in physical education classes with good tolerance; however, she avoids prolonged and intensive physical exertion. She remains under multidisciplinary care and continues to undergo rehabilitation.

## Discussion

Interventricular septal hematoma is an uncommon but potentially fatal postoperative complication, with mortality rates reaching up to 40% particularly in patients presenting with severe hemodynamic instability or malignant arrhythmias (1, 2). Extracorporeal membrane oxygenation may stabilize circulation in the acute phase when low cardiac output is related to rhythm disturbances or transient myocardial dysfunction (4). However, prolonged systemic anticoagulation may interfere with intralesional thrombus formation.

The limited number of reported cases precludes development of evidence-based management recommendations (1, 2). Treatment strategies therefore remain empirical and individualized.

Management of interventricular septal hematoma in small infants is particularly challenging. In the absence of ventricular communication most lesions undergo spontaneous resorption within weeks and can be safely managed conservatively. When communication is present, interventional strategies may not eliminate the bleeding source and may increase the risk of irreversible injury to the atrioventricular conduction system.

Available data on interventricular septal hematoma are derived predominantly from small case series and isolated reports, most of which describe spontaneous regression within weeks to a few months (2, 7). Long-term follow-up data remain scarce, with the longest reported observation of approximately 6 years described by Zhang et al. (8).

In the present patient, currently 10 years old, any attempt to close communication between the LV cavity and the hematoma remains highly controversial due to risk of AV block and possible recurrent hematoma expansion. The prolonged observation period provides insight into the natural history of this rare entity and demonstrates long-term structural stability and preserved ventricular function under conservative management.

Despite structural stability, thromboembolic complications may occur, as illustrated by ischemic stroke observed in this patient, rendering long-term anticoagulation necessary.

Low-dose aspirin is commonly used as antiplatelet thromboprophylaxis in children with congenital heart disease, particularly after surgical or catheter-based interventions and in the presence of prosthetic material or altered intracardiac flow. Current pediatric antithrombotic recommendations suggest aspirin doses of 1–5 mg/kg/day, with commonly used regimens of 3–5 mg/kg/day and a maximum daily dose of 75 mg (5, 6). In the absence of evidence-based recommendations for postoperative interventricular septal hematoma, the initial use of low-dose aspirin in our patient was consistent with the routine postoperative antithrombotic practice at our institution for children without an established indication for systemic anticoagulation. Our treatment strategy was based on balancing the theoretical risk of thromboembolism against the potential risk of recurrent intramyocardial bleeding.

Nevertheless, in our patient, ischemic stroke occurred despite ongoing antiplatelet therapy, indicating failure of low dose aspirin-based thromboprophylaxis in the setting of a persistent high-risk intracardiac substrate. This raises the question of whether a higher aspirin dose or earlier initiation of anticoagulation therapy might have prevented the observed complication.

Consequently, the therapeutic strategy was reconsidered. Following the ischemic stroke, the antithrombotic strategy was reassessed by a multidisciplinary team, including pediatric neurologists and hematologists. Based on the presumed cardioembolic mechanism and the persistent communication between the interventricular septal hematoma and the left ventricular cavity, long-term oral anticoagulation with warfarin was initiated, with a target INR of 2.0–3.0.

The ischemic stroke prompted consideration of invasice closure of the communication between the left ventricular cavity and the interventricular septal hematoma. After multidisciplinary consultations involving several tertiary congenital heart disease centers both nationally and internationally, the procedure was deferred because of the large size of the lesion and the anticipated technical challenges associated with intervention at the patient's age. It was agreed to postpone potential closure until adolescence. The patient is currently undergoing further diagnostic evaluation to determine the feasibility and optimal timing of the planned intervention.

## Conclusions

This case documents the longest reported follow-up (10 years) of a giant postoperative interventricular septal hematoma with persistent communication to the left ventricular cavity following surgical closure of a perimembranous ventricular septal defect. Although long-term structural stability and preserved ventricular function were maintained despite the absence of hematoma regression, persistent communication with the left ventricular cavity may remain a source of late thromboembolic complications. Therefore, conservative management alone may not be sufficient in all patients, and treatment should be individualized based on multidisciplinary assessment, incorporating long-term imaging surveillance, optimization of antithrombotic therapy, and periodic reassessment of the potential need for invasive intervention.

### Learning Points

- Persistent communication between the hematoma cavity and the LV may remain stable during long-term follow-up.
- Conservative management may be associated with preserved ventricular function in the absence of hemodynamic compromise.
- Late thromboembolic complications may occur despite clinical stability and antiplatelet therapy.
- Serial echocardiography and cardiac magnetic resonance imaging are valuable for long-term surveillance.

## Data Availability

The original contributions presented in the study are included in the article/Supplementary Material, further inquiries can be directed to the corresponding author.

## Supplementary material

Supplementary Video 1–3 Immediate postoperative transthorasic echokardiography showing a large ventricular septal hematoma protruding into both vantricular cavieties without compromise of the inflow or outflow tracts.

Supplementary Video 3–4 Early postoperative transthoracic echocardiography showing the interventricular septal hematoma without detectable communication with the ventricular cavities.

Supplementary Video 4 Early postoperative transthoracic echocardiography showing the interventricular septal hematoma and a small residual ventricular septal defect.

Supplementary Video 5 Transthoracic echocardiography one month after surgery showing multiple small fistulous communications between the hematoma cavity and the left vntricular lumen predominantly in the apical region. The Conflict of Interest statement is correct.

Supplementary Video 6 Cardiac magnetic resonance imaging at 7 months of age showing a large blood-filled cavity involving the basal, mid, and apical segments of the interventricular septum, with persistent communication with the left ventricular cavity at the apical level.

Supplementary Video 7–9 Follow-up transthoracic echocardiography demonstrating stable morphology of the interventricular septal hematoma.

Supplementary Video 10 Follow-up cardiac magnetic resonance imaging performed at 5 years demonstrating stable morphology of the interventricular septal hematoma.

Supplementary Video 11 Follow-up cardiac magnetic resonance imaging performed at 9 years demonstrating stable morphology of the interventricular septal hematoma.
